# An Examination of the Effect of Parent‐Centered Nutrition Education on the Oxidant‐Antioxidant Parameters of Children Diagnosed With Autism Spectrum Disorder

**DOI:** 10.1002/brb3.70504

**Published:** 2025-05-13

**Authors:** Diler Us Altay, Erman Esnafoglu, Emine Kocyigit, Duygu Mataraci Değirmenci, Tevfik Noyan

**Affiliations:** ^1^ Faculty of Health Sciences, Department of Nutrition and Dietetics Ordu University Ordu Turkey; ^2^ Faculty of Medicine, Department of Child and Adolescent Mental Health Dısorders Ordu University Ordu Turkey; ^3^ Faculty of Medicine, Department of Medical Biochemistry Ordu University Ordu Turkey

**Keywords:** antioxidant, autism spectrum disorder, nutrition education, oxidant

## Abstract

**Background:**

This study examined the effect of nutrition education given by dietitians to families of children aged 3–18 diagnosed with autism spectrum disorder (ASD) on meal consumption, eating behaviors, autism severity, serum oxidant/antioxidant marker levels, and total dietary antioxidant capacity.

**Methods:**

The project was carried out with 44 pediatric patients diagnosed with ASD and their parents. The ELISA method was used for antioxidant and oxidant measurements, and the oxygen radical absorbance capacity values ​​of foods defined according to the BeBiS program were used to calculate the total dietary antioxidant capacity. The children's eating behavior questionnaire, childhood autism rating scale (CARS), and brief autism mealtime behavior inventory (BAMBI) were administered.

**Results:**

There was no significant difference in antioxidant and oxidant parameters between the experimental and control groups. At the end of eight weeks, superoxide dismutase and glutathione levels increased significantly in the experimental group. There was no significant difference in terms of the families of the autistic children in the experimental and control groups or their disease‐specific knowledge. BAMBI scores were similar between the groups at baseline, while a significant decrease was observed in the experimental group at the end of the study. Daily energy, saturated fatty acid (SFA), carbohydrate, and omega‐6 intake decreased, while protein, fat, mono‐ and poly‐unsaturated fatty acid, and omega‐3 intake increased in the experimental group following nutrition education. However, these results were not statistically significant. There was no significant difference in terms of micronutrient intake between children with ASD in experimental and control groups before and after nutrition education.

**Conclusion:**

Improvements in eating habits and dietary patterns were noted after nutrition education, especially in the experimental group, even though there were no appreciable changes in oxidative stress indicators. These behavioral shifts imply that family nutrition education can be extremely important in encouraging better eating practices and improving the general well‐being of kids with ASD and their families.

## Graphical abstract

Autism spectrum disorder (ASD) is a neurodevelopmental disorder that produces symptoms from the early years of life. This study was conducted to examine the effect on total antioxidant capacity and antioxidant–oxidant parameters in the diet in children of nutrition education provided by specialist dieticians for the families of children diagnosed with ASD aged 3–18 years. As a result, it shows that the nutrition education provided helps children with ASD acquire healthy nutrition and eating habits, prevents nutritional deficiencies, alleviates nutrition‐related symptoms, ensures healthy growth and development, improves the quality of life of families, and reduces stress factors.

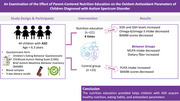

## 1 Introduction

Autism spectrum disorder (ASD) is a neurodevelopmental disorder that produces symptoms from the early years of life and is characterized by inadequacy in communication and social interaction, repetitive movements, and limited fields of interest and activity (Esnafoğlu 2017). Symptoms of autism are generally recognized in the first three years and persist throughout life (Chen et al. 2015). Recent epidemiological studies have shown that the prevalence is increasing, and although the exact figure in Türkiye is uncertain, it is estimated to be close to that in the rest of the world (Çağlar and Özkan 2021). The pathogenesis of the disease is also not fully understood. Although the etiology is associated with several genetic and environmental factors, genetic causes have been reported to be more prominent, with antioxidant defense against increasing oxidative stress decreasing and the oxidative balance being impaired. Other causes such as immune deficiencies, toxic substances, and infections are regarded as environmental factors (Chaste and Lebover 2022. Nutrition is another powerful environmental risk factor (Dufault et al. 2023). A bi‐directional relationship exists between autism and nutrition. While foodstuffs thought to cause autism are currently under discussion, it has also been suggested that specific foods can prevent the development and progression of the condition. Some foods can adversely affect brain development and give rise to autistic behaviors (Doreswamy et al. 2020). In addition, eating disorders such as excessive food selectivity, ritual eating behaviors, food rejection, and food aversion are frequently seen in children with ASD (Şengüzel et al. 2021; Yeung et al. 2021). Nutritional components (such as vitamins, minerals, and amino acids) involved in neurotransmitter synthesis and enzyme activation must be consumed regularly and in a balanced manner to improve brain functions in children with ASD. Research has identified probiotics, enzymes, vitamins, and minerals as key compounds in the biomedical approach to autism. At the same time, various dietary interventions have played an effective role in improving ASD symptoms. While ASD was once thought to be a solely psychological or neurological disease, there is now growing evidence that it affects several systems (metabolic, gastrointestinal, immunological, mitochondrial, neurological, etc.). Gastrointestinal system symptoms and eating/nutrition problems are comorbidities frequently seen in ASD. Dufault et al. (2021) examined the link between blood levels of inorganic mercury and lead and their dietary sources, emphasizing how these toxic metals might affect child development and potentially be associated with neurodevelopmental disorders, such as ASD. They suggested that these metals could serve as biomarkers in children and may play a role in ASD. Diagnosis relies on clinical evaluation and behavioral characteristics, although symptoms vary from one individual to another. There is also no drug available for symptoms associated with ASD. However, several drugs are employed to help manage concentration problems, depression, and tantrums. Skills training programs and psychosocial interventions represent the basis of treatment (Guthrie et al. 2013). The aim of treatment is to increase the independent living skills of individuals with ASD, strengthen their social communication and interaction, expand narrow fields of interest, and reduce potentially disturbing behaviors to a minimum. Some patients have benefitted from such treatment, although others have occasionally exhibited various complications. The main treatment for ASD is an appropriate educational approach. The inclusion of parents in this education program is of very great importance. The objective of educational therapies is the improvement of social communication and interaction and the acquisition of new skills.

Antioxidants are beneficial in protecting against autism and play a vital role in ameliorating damage caused by increasing oxidative stress and free radicals. Foods and components thereof with antioxidant properties are therefore highly important in protecting the body against oxidative stress (Chauhan et al. 2004; Sen and Chakraborty 2011; Bjørklund et al. 2020). Approximately 90% of children with ASD experience problems with nutrition (Ledford and Gast 2006). Parents report that children with ASD develop high levels of allergies to milk and milk products, fatty seeds, and fruits (Lyall et al. 2015; Gurney et al. 2006). Selective eating, food rejection, and fussiness are problematic eating behaviors frequently reported in children with ASD. Such children generally select food based on their color, shape, structure, or temperature (Postorino et al. 2015). The rejection of fruits, vegetables, and protein‐rich foods encourages children with ASD to consume processed foods with high energy and carbohydrate content. Increased consumption of snacks and high‐energy foods leads to an increase in body weight among children with ASD, and rates of obesity in children with the disorder and diseases such as diabetes and hypertension that develop due to obesity in later life are higher than those in healthy children (Sharp et al. 2013; Curtin et al. 2010). Nutrition education that provides individuals with knowledge and information and that enables them to make informed decisions about their nutrition is used to encourage healthy nutrition in society (Yeung et al. 2021; Uribe and Olson 2020).

The family is highly influential in the child's correct acquisition of eating habits. Children with ASD need their parents to meet their nutritional requirements (Dufault et al. 2024). Meral (2017) reported that children with ASD develop eating disorders more frequently than children with normal development and that their families are more resistant to consuming different food groups and experience greater difficulty on the subject. Children with ASD develop behaviors involving the consumption of specific foods at mealtimes, so that food diversity cannot be provided, because of which parents and others responsible for children's care may become stressed (Aponte and Romanczyk 2016). Family‐centered nutrition is reported to have a positive impact on eating behavior and to reduce food selectivity among children with ASD (Manzanarez et al. 2021; Sharp et al. 2019). Research has concluded that the psychosocial and nutritional behaviors of individuals with ASD (aged 12–20) can be improved by means of online virtual nutrition education (Buro et al. 2022). The number of studies regarding the nutrition status of children with autism is limited. However, the link between nutrition and the etiology and course of autism should not be overlooked, and the nutritional status of individuals with autism should be considered. No previous studies have evaluated the effect of parent‐centered nutrition education on the total antioxidant capacity of diet and the effect on antioxidant and oxidant parameters in response to increased oxidative stress and decreased antioxidant defense in autism. This study was conducted to examine the effect of total antioxidant capacity and antioxidant–oxidant parameters in the diet in children of nutrition education provided by specialist dieticians for the families of children diagnosed with ASD aged 3–18 years.

## 2 Material and Method

The research population consisted of patients with ASD presenting to the Ordu University Education and Research Hospital Child and Adolescent Mental Health and Diseases Clinic in the Turkish province of Ordu. The research sample consisted of 44 patients, and the parents thereof, presenting to the clinic and diagnosed with ASD based on DSM‐5 criteria as a result of clinical psychiatric examination and assessment performed by a specialist physician, and who met the inclusion criteria and consented to take part.

Homogeneity of the study population was important, and every effort was made to ensure there was no significant difference between the groups (in terms of age, sex, and body mass index [BMI]) for age *z* scores. The parents of the autistic children were divided into two groups, study and control, depending on whether they received nutrition education. The research involved two stages, pre‐education (Day 0, first interview) and post‐nutrition education (Week 8). The first (study) group consisted of 21 ASD patients whose parents were given nutrition education, and the second (control) group, of 23 ASD patients without nutrition education. The inclusion criteria were diagnosis of ASD based on DSM‐5 criteria, age 3–18 years, routine tests being normal, and BMI for age *z* score cut‐off points being ≥−1 SD ≤ 1 SD (normal).

The exclusion criteria were a history of systemic disease, neurological diseases (cerebral palsy, epilepsy, sensory defects, motor deficit, or tumor), abnormality on routine tests (thyroid function, liver function, and kidney function tests), presence of acute or chronic infection, the use of any pharmacological agent in the previous six months, receipt of nutritional support in the previous six months, use of antioxidants, supplements, or similar), BMI for age cut‐off points being severely thin (<‐2 SD), thin (≥‐2 SD<‐1 SD), slightly obese (>1 SD), or obese (>2 SD), and presence of metabolic or endocrine diseases.

The research data were collected using a questionnaire administered by the researchers at face‐to‐face interviews. This was read by the researchers and completed with the parents (mother or father) of the children taking part in the study. It consisted of seven sections. The first contained questions investigating general information concerning the children with ASD and their parents (sex, age, number of siblings, education and receipt of special education, age, occupation, and education level of mother and father), and the second was designed to elicit the autistic children's health details. The third section involved anthropometric measurements and body mass index, the fourth consisted of the childhood autism rating scale (CARS), the fifth and sixth of the brief autism mealtime behavior inventory (BAMBI), and children's eating behavior questionnaire, administered twice to the study and control groups (on Weeks 0 and 8), the seventh of a food consumption record form and a determination of total diet antioxidant capacity, and the eighth of biochemical findings. The parents included in the study group received nutrition education four times (over two months) at two‐week intervals, each session lasting at least 40 min, using the face‐to‐face interview technique administered by specialist dieticians. This study determined, for the first time in literature, whether the specified period would result in changes in the biochemical measurements of patients diagnosed with ASD and receiving or not receiving nutrition education.

Researchers conducted a thorough literature review to gather information necessary for developing a nutrition education intervention tailored to specific behaviors and sensitive to cultural factors. The nutrition education for children with ASD included the relationship between autism and nutrition, daily energy and nutrient intake of children with ASD, identification of food groups, food selectivity, food rejection, gastrointestinal issues, and treatment of nutrient deficiencies, as well as the development of strategies for other challenges faced in the nutrition (Matthews and Adams 2023; Önal et al. 2023; Arija et al. 2023). Education 1 focused on the relationship between ASD and nutrition, the importance of healthy nutrition in ASD, gaining healthy eating habits, problems affecting the nutrition of children with ASD, determining the child's attitudes and behaviors towards food, and exploring various dietary interventions that may support better health outcomes. Education 2 emphasized the daily energy and nutrient requirements (protein, carbohydrate, fat, vitamins, minerals, and water) for children with ASD, the significance of main meal and snack consumption, healthy and safe food selections, the definition of food groups (milk and dairy products, meat and meat products, legumes and oil seeds, vegetables and fruits, cereals), the importance of consuming these food groups, and the assessment of daily food group requirements. The significance of foods rich in omega‐3 fatty acids (such as fish and walnuts) and the necessity of reducing the intake of omega‐6 sources (like sunflower and maize oil). Education 3 concentrated on knowledge of food selectivity, food rejection, gastrointestinal symptoms, and strategies for obesity prevention through the elimination of dietary deficits and the enhancement of physical activity. Education 4 emphasized devising solutions for further obstacles faced in the nutrition of children with ASD, recognizing the significance of sustainable nutrition, and providing menu samples. Education was given face‐to‐face, four times and for 1 h, for eight weeks.

Routine biochemical findings from the patients with ASD enrolled in the study and control groups (complete blood count, biochemical analyses including liver and kidney function tests, thyroid function tests, inflammatory markers such as C‐reactive protein and sedimentation, vitamins B12 and D, folate, iron and iron binding, and ferritin) were retrieved from the patients’ records on Weeks 0 and 8 and recorded. In addition, glutathione (GSH), catalase (CAT), malondialdehyde (MDA), 8‐hydroxydeoxyguanosine (8‐OHdG), and superoxide dismutase (SOD) levels were measured using the ELISA method for the measurement of serum oxidant/antioxidant levels.

The data obtained were analyzed using the Statistical Package for the Social Sciences (SPSS, version: 22.0) for Windows software. After the study, the results obtained were expressed as arithmetical mean +standard deviation (x̄±SD) and mean plus interquartile range. Data compatibility with normal distribution was assessed using visual methods (histogram and probability charts) and analytical methods (the Kolmogorov–Smirnov/Shapiro–Wilk tests). Spearman/Pearson correlation tests were applied to evaluate relationships between different parameters, while categorical variables were compared between the groups using the chi‐square test. *α* = 0.05 was selected as the error level for all analyses, with *p* values equal to or smaller than that value being regarded as statistically significant.

## 3 Results

An evaluation of the antioxidant and oxidant parameters of the patients diagnosed with autism is shown in Table 1. There was no significant difference between the experimental and control groups in terms of GSH, MDA, SOD, CAT, or 8‐OHdG values. At the end of the eighth week, SOD, 8‐OHdG, and GSH levels increased significantly in the experimental group receiving nutrition education intervention.

**TABLE 1:** Antioxidant–oxidant serum parameters.

Serum parameters		Experimental group (*n* = 21)	Control group (*n* = 23)	*p* value
SOD (pg/mL)	Pre‐test	634.6 (633.6–635.1)	634.3 (633.6–635.1)	*Z* = −0.215 *p* = 0.830
	Post‐test	635.6 (634.3–635.7)	635.6 (634.1–636.3)	*Z* = −0.120 *p* = 0.904
		*Z* = −2.376 *p* = 0.018	*Z* = −1.254 *p* = 0.210	
CAT (U/mL)	Pre‐test	174.8 (115.3–196.0)	186.0 (143.7–215.7)	*Z* = −1.291 *p* = 0.197
	Post‐test	177.6 (125.2–203.8)	200.8 (161.9–221.0)	*Z* = −0.904 *p* = 0.366
		*Z* = −0.795 *p* = 0.426	*Z* = −0.698 *p* = 0.485	
MDA (ng/mL)	Pre‐test	234.1 (160.8–390.8)	230.0 (169.6–289.1)	*Z* = −0.014 *p* = 0.839
	Post‐test	316.5 (175.4–394.2)	213.2 (157.8–289.8)	*Z* = −1.512 *p* = 0.131
		*Z* = −0.220 *p* = 0.826	*Z* = −0.485 *p* = 0.627	
8‐dOH (ng/mL)	Pre‐test	26.1 (18.5–48.9)	42.5 (30.7–64.3)	*Z* = −1.843 *p* = 0.065
	Post‐test	49.6 (38.4–85.1)	54.8 (37.3–73.9)	*Z* = −0.075 *p* = 0.940
		*Z* = −2.207 *p* = 0.027	*Z* = −2.172 *p* = 0.030	
GSH (µg/mL)	Pre‐test	29.4 (20.9–55.4)	48.0 (34.7–72.7)	*Z* = −1.843 *p* = 0.065
	Post‐test	56.1 (43.4–96.2)	62.1 (42.2–83.6)	*Z* = −0.075 *p* = 0.940
		*Z* = –2.207 *p* = 0.027	*Z* = –2.190 *p* = 0.029	

*Note*: Median (IQR); Independent parameters, Mann–Whitney *U* test; Dependent parameters, Wilcoxon test.

Nonparametric *p* < 0.05

Information concerning the families of the autistic children is given in Table 2. The mean age of the participants’ mothers was 35.30 ± 5.67, and the mean age of the fathers was 39.41 ± 6.59. Analysis showed that 43.2% of mothers and 50% of fathers were high school graduates. Only 2% of mothers and 90.9% of fathers were in regular employment. In addition, 50% of the children had single siblings, and 38.6% had two siblings. No significant difference was observed between the two groups in terms of familial characteristics (*p *> 0.05).

**TABLE 2:** Familial characteristics.

	All participants (*n* = 44)	Experimental group (*n* = 21)	Control group (*n* = 23)	*p*	t/χ2
	*n* (%)	*n* (%)	*n* (%)		
Mothers’ age (years χ̅ ± SD)	35.30 ± 5.67 35.00(23.0–51.0)	35.55 ± 6.93 35.50(23.0–51.0)	35.09 ± 4.45 35.0(27.0–45.0)	0.793^a^	0.264
Fathers’ age (years χ̅ ± SD)	39.41 ± 6.59 38.50(27.0–57.0)	38.80 ± 7.87 38.0(27.0–57.0)	39.95 ± 5.29 39.0(31.0–53.0)	0.571^a^	−0.572
Mothers’ education status					
Literate	1 (2.3%)	1 (4.8%)	—	0.088^b^	9.578
Elementary school	6 (13.6%)	3 (14.3%)	3 (13.0%)		
Middle school	12 (27.3%)	8 (38.1%)	4 (17.4%)		
High school	19 (43.2%)	7 (33.3%)	12 (52.2%)		
University	4 (9.1%)	—	4 (17.4%)		
Postgraduate	2 (4.5%)	2 (9.5%)	—		
Fathers’ education status					
Literate	1 (2.3%)	1 (4.8%)	—	0.496^b^	4.379
Elementary school	6 (13.6%)	3 (14.3%)	3 (13.0%)		
Middle school	3 (6.8%)	2 (9.5%)	1 (4.3%)		
High school	22 (50.0%)	9 (42.9%)	13 (56.5%)		
University	10 (22.7%)	4 (19.0%)	6 (26.1%)		
Postgraduate	2 (4.5%)	2 (9.5%)	—		
Mothers’ total length of education (years χ̅ ± SD)	10.11* ± *3.49 11.0(0.00‐18.0)	9.45* ± *4.01 9.0(0.0‐18.0)	10.69* ± *2.94 11.0(5.0‐16.0)	0.214^a^	−1.263^a^
Fathers’ total length of education (years χ̅ ± SD)	11.06* ± *3.81 11.0(0.0‐18.0)	10.80* ± *4.47 11.0(0.0‐18.0)	11.30* ± *3.18 11.0(5.0‐16.0)	0.673^a^	−0.425
Mother's wok status					
Working	2 (4.5%)	2 (9.5%)	—	0.130^b^	2.295
Not working	42 (95.5%)	19 (90.5%)	23 (100%)		
Fathers’ work status					
Working	40 (90.9%)	18 (85.7%)	22 (95.7%)	0.252^b^	1.312
Not working	4 (9.1%)	3 (14.3%)	1 (4.3%)		
Mothers’ income levels (Euros χ̅ ± SD)	132.27* ± *316.53 0.0(0.0–1058.2)	252.52* ± *428.44 0.0(0.0–1058.2)	833.33* ± *2886.75 0.0(0.0–10000.0)	0.456^a^	0.854
Fathers’ income levels (Euros χ̅ ± SD)	871.1* ± *781.32 661.4(132.3–5291.0)	769.98* ± *1122.54 529.10(132.27–5291.0)	3954.68* ± *300.99 1058.20(529.10–1455.02)	0.453^a^	0.174
Number of siblings					
1	22 (50%)	10 (47.6%)	12 (52.2%)	0.541^b^	2.154
2	17 (38.6%)	9 (42.9%)	8 (34.8%)		
3	4 (9.1%)	1 (4.8%)	3 (13.0%)		
4	1 (2.3%)	1 (4.8%)	—		

^a^ *p*: Independent sample *t* test; ^b^ *p*: Pearson's chi‐square test.

Demographic and disease‐related data for the autistic children in the study are shown in Table 3. These were similar between the two groups (*p* > 0.05). Analysis showed that 81.8% of the participants consumed three main meals a day and that 93.2% applied no special diet. Evaluation of the CARS scores showed that mild‐moderate autism was present in 27.3% of the participants, and severe autism in 72.7%. No significant difference in autism severity or scale scores was observed between the groups (*p* > 0.05).

**TABLE 3:** Child‐ and disease‐related data.

	All participants (*n* = 44)	Experimental group (*n* = 21)	Control group (*n* = 23)	*p*	t/U/χ2
	*n* (%)	*n* (%)	*n* (%)		
Age	6.26* ± *1.96 6.0 (3.0–11.0)	5.80* ± *1.80 5.0(3.0–10.0)	6.67* ± *2.05 6.0 (4.0–11.0)	0.147^a^	−1.476
Gender					
Male	20 (45.5%)	10 (47.6%)	10 (43.5%)	0.783^c^	0.076
Female	24 (54.5%)	11 (52.4%)	13 (56.5%)		
Regular medication use					
Yes	19 (43.2%)	12 (57.1%)	7 (30.4%)	0.074^c^	3.191
No	25 (56.8%)	9 (42.9%)	16 (69.6%)		
Number of main meals consumed					
1	1 (2.3%)	1 (4.7%)	—	0.558^c^	1.165
2	7 (15.9%)	3 (14.3%)	4 (17.4%)		
3	36 (81.8%)	17 (81.0%)	19 (82.6%)		
Number of snack meals consumed					
0	9 (20.5%)	2 (9.5%)	7 (30.4%)	0.296^c^	3.695
1	8 (18.2%)	4 (19.0%)	4 (17.4%)		
2	18 (40.8%)	9 (42.9%)	9 (39.2%)		
3	9 (20.5%)	6 (28.6%)	3 (13.0%)		
Type of delivery					
Vaginal route	15 (34.1%)	9 (42.9%)	6 (26.1%)	0.241^c^	1.374
Cesarean	29 (65.9%)	12 (57.1%)	17 (73.9%)		
Duration of breastfeeding (months χ̅ ± SD)	14.51* ± *11.98 10.0(0.0–36.0)	18.0* ± *13.16 14.0(1.0–36.0)	11.32* ± *10.03 8.0(0.0–36.0)	0.064^a^	1.901
Age at diagnosis (years χ̅ ± SD)	2.71* ± *1.26 2.75(0.75–9.0)	2.50* ± *0.67 2.50(1.0–3.5)	2.90* ± *1.62 3.0(0.75–9.0)	0.569^b^	218.000
Special diet					
Yes	3 (6.8%)	3 (14.3%)	—	0.060^c^	3.526
No	41 (93.2%)	18 (85.7%)	23 (100%)		
Use of food supplements					
Yes	14 (31.8%)	10 (47.6%)	4 (17.4%)	0.068^c^	3.335
No	30 (68.2%)	11 (52.4%)	19 (82.6%)		
Receipt of special education					
Yes	39 (88.6%)	16 (76.2%)	23 (100%)	0.013*^c^	6.178
No	5 (11.4%)	5 (23.8%)	—		
Body weight (kg χ̅ ± SD)	24.45* ± *7.02 23.0(14.0–51.0)	23.28* ± *6.51 22.5(14.0–36.0)	25.52* ± *7.45 23.0(18.0–51.0)	0.258^b^	193.500
Height (cm χ̅ ± SD)	120.36* ± *12.98 120.50(95.0–148.0)	118.33* ± *14.05 120.0(95.0–138.0)	122.21* ± *11.92 121.0(102.0–148.0)	0.327^a^	−0.991
Childhood Autism Rating Scale Score	40.15* ± *6.18 41.0 (25.50–52.0)	41.92* ± *5.88 43.0 (31.0–52.0)	38.54* ± *6.12 38.0 (25.5–50.5)	0.069^a^	1.866
Degree of autism					
Mild‐moderate (scores 30–36.5)	12 (27.3%)	4 (19.0%)	8 (34.8%)	0.242^c^	1.370
Severe (scores 37–60)	32 (72.7%)	17 (81.0%)	15 (65.2%)		

^a^ *p*: Independent sample *t* test; ^b^ *p*: Mann–Whitney *U* test; ^c^ *p*: Pearson's chi‐square test.

Table 4 shows information regarding the eating behaviors of children with autism. At the beginning of the study, no significant differences were observed between the groups in terms of BAMBI, children's eating behavior questionnaire, and their subscale scores (*p* > 0.05). Higher Bambi scores indicate more negative behaviors specific to autism. As expected, a significant decrease in BAMBI total and subscale scores (limited variety, food refusal, and features of autism) in the experimental group (*p* < 0.05), and a significant increase in the control group (*p* < 0.05). A significant decrease was observed in the experimental group in children's eating behavior questionnaire total scores and food responsiveness, desire to drink, and satiety responsiveness subscale scores (*p* < 0.05). In the control group, statistically significant increases were determined in children's eating behavior questionnaire total scores and food responsiveness, emotional overeating, enjoyment of food, satiety responsiveness, slowness in eating, emotional undereating, and fussiness subscale scores (*p* < 0.05). However, see Table 4 for the exact *p* values of the variables.

**TABLE 4:** Eating behavior characteristics of children with autism.

		Experimental group (*n* = 21)	Control group (*n* = 23)	*p*	*t*
Brief autism mealtime behavior inventory (BAMBI) total score	Pre‐test	47.90 ± 9.29 50.0(20.0–61.0)	47.60 ± 11.57 51.0(22.0–63.0)	0.926^a^	0.093
	Post‐test	43.76 ± 8.30 46.0 (19.0–55.0)	49.26 ± 12.75 54.0 (22.0–65.0)	0.096^a^	−1.708
	p	< 0.001^b^*	0.001^b^*		
	t	7.213	−3.731		
Subscales of BAMBI					
Limited food variety	Pre‐test	26.09 ± 5.58 28.0(10.0–34.0)	25.30 ± 6.58 28.0(12.0–34.0)	0.671^a^	0.427
	Post‐test	24.14 ± 5.33 25.0(9.0–32.0)	25.69 ± 6.85 29.0(12.0–34.0)	0.409^a^	−0.833
	p	< 0.001^b^*	0.009^b^*		
	t	5.499	−2.859		
Food refusal	Pre‐test	10.90 ± 3.16 11.0(5.0–16.0)	11.26 ± 3.88 10.0(5.0–18.0)	0.742^a^	−0.331
	Post‐test	9.85 ± 2.39 10.0(5.0–14.0)	11.82 ± 4.0(11.0(5.0–18.0)	0.053^a^	−1.998
	p	0.001^b^*	0.012^b^*		
	t	3.740	−2.732		
Features of autism	Pre‐test	10.90 ± 2.93 12.0(5.0–16.0)	11.04 ± 2.82 11.0(5.0–16.0)	0.874^a^	−0.160
	Post‐test	9.76 ± 2.40 10.0(5.0–13.0)	49.26 ± 12.75 54.0(22.0–65.0)	0.025^a^*	−2.329
	p	< 0.001^b^*	0.010^b^*		
	t	5.435	−2.816		
**Children's eating behavior questionnaire total score**	Pre‐test	94.57 ± 14.85 97.0(68.0–118.0)	94.95 ± 12.24 97.0(76.0–118.0)	0.925^a^	−0.094
	Post‐test	90.52 ± 12.19 94.0(69.0–108.0)	99.65 ± 15.81 103.0(76.0–125.0)	0.039^a^*	−2.129
	p	0.004^b^*	< 0.001^b^*		
	t	3.222	−4.421		
**Subscales of children's eating behavior questionnaire**					
Food responsiveness	Pre‐test	12.61 ± 4.92 13.0(5.0–22.0)	12.86 ± 6.57 13.0(5.0–25.0)	0.888^a^	−0.142
	Post‐test	12.04 ± 4.62 11.0(5.0–20.0)	13.60 ± 7.10 15.0(5.0–25.0)	0.389^a^	−0.871
	p	0.030^b^*	0.001^b^*		
	t	2.335	−3.872		
Emotional overeating	Pre‐test	6.71 ± 2.05 7.0(4.0–11.0)	6.95 ± 2.03 8.0(4.0–12.0)	0.696^a^	−0.393
	Post‐test	6.57 ± 1.59 7.0(4.0–9.0)	7.17 ± 2.18 8.0(4.0–12.0)	0.300^a^	−1.049
	p	0.526	0.022^b^*		
	t	0.645	−2.472		
Enjoyment of food	Pre‐test	15.76 ± 6.35 17.0(6.0–25.0)	15.69 ± 5.67 15.0(7.0–25.0)	0.971^a^	0.037
	Post‐test	15.33 ± 5.91 17.0(6.0–25.0)	16.39 ± 5.51 17.0(7.0–25.0)	0.542^a^	−0.614
	p	0.225	0.008^b^*		
	t	1.253	−2.912		
Desire to drink	Pre‐test	9.95 ± 4.24 11.0(3.0–15.0)	8.60 ± 4.83 9.0(3.0–15.0)	0.335^a^	0.976
	Post‐test	9.0 ± 3.57 10.0(3.0–14.0)	8.95 ± 4.96 10.0(3.0–15.0)	0.973^a^	0.034
	p	0.003^b^*	0.057		
	t	3.400	−2.006		
Satiety responsiveness	Pre‐test	21.80 ± 5.06 21.0(11.0–32.0)	20.86 ± 5.26 20.0(12.0–31.0)	0.550^a^	0.602
	Post‐test	20.61 ± 4.26 21.0(12.0–29.0)	21.56 ± 5.0 21.0(12.0–31.0)	0.505^a^	−0.672
	p	0.004^b^*	0.003^b^*		
	t	3.283	−3.272		
Slowness in eating	Pre‐test	10.71 ± 4.10 10.0(4.0–17.0)	10.86 ± 4.28 10.0(4.0–17.0)	0.903^a^	−0.122
	Post‐test	10.33 ± 3.78 11.0(4.0–16.0)	11.65 ± 4.35 12.0(4.0–18.0)	0.292^a^	−1.067
	p	0.268	0.011^b^*		
	t	1.139	−2.787		
Emotional undereating	Pre‐test	9.33 ± 2.95 8.0(4.0–15.0)	10.39 ± 2.21 10.0(8.0–15.0)	0.183^a^	−1.352
	Post‐test	9.09 ± 2.93 8.0(4.0–15.0)	10.95 ± 1.94 10.0(8.0–15.0)	0.019^a^*	−2.459
	p	0.096	0.002^b^*		
	t	1.746	−3.441		
Fussiness	Pre‐test	7.66 ± 3.46 8.0(3.0–12.0)	8.69 ± 3.32 10.0(3.0–15.0)	0.321^a^	−1.005
	Post‐test	7.52 ± 3.07 8.0(3.0–12.0)	9.34 ± 3.44 10.0(3.0–15.0)	0.072^a^	−1.845
	p	0.480	0.001^b^*		
	t	0.719	−3.761		

*p* ^a^: Independent sample *t* test; *p* ^b^: Paired sample *t* test.

The children's mean energy, macro, and micronutrient intakes before and after nutrition education are shown in Tables 5 and 6. The omega‐6/omega‐3 value of the children in the experimental group decreased significantly between Week 0 and Week 8 (*p* < 0.05), while no significant difference was observed in other energy and macronutrient values (*p* > 0.05). Polyunsaturated fatty acids (PUFA) intake in the children in the control group increased by Week 8 (*p* < 0.05), while no significant difference was observed in other energy and macronutrient values (*p *> 0.05). Examination of the effect of nutrition education on energy and macronutrient intakes between the two groups showed an increase in monounsaturated fatty acids (MUFA) (%) and a decrease in daily dietary fiber intake in the children from the control group (*p *< 0.05). No statistically significant difference was determined within and between the groups in terms of micronutrient intake before and after nutrition education (*p* > 0.05).

**TABLE 5:** Energy and macro nutrient intakes and total dietary antioxidant capacities of autistic children before and after nutrition education.

	Experimental group (*n* = 21)			Control group (*n* = 23)			
	Week 0	Week 8	^a^ *p*	Week 0	Week 8	^b^ *p*	^c^ *p*
	Mean±SD	Mean±SD		Mean±SD	Mean±SD		
Energy intake (kcal/day)	1238.6 ± 328.65	1192.5 ± 239.4	0.772	1156.8 ± 382.84	1146.3 ± 332.76	0.742	0.192
Protein (%)	14.0 ± 3.00	15.0 ± 3.00	0.147	15.0 ± 3.00	15.0 ± 2.00	0.468	0.637
Fat (%)	37.0 ± 6.00	38.0 ± 10.00	0.555	43.0 ± 8.00	43.0 ± 7.00	0.966	0.094
SFA (%)	14.6 ± 3.22	14.2 ± 4.17	0.681	17.2 ± 4.30	16.4 ± 4.45	0.536	0.093
MUFA (%)	12.8 ± 2.95	12.9 ± 4.33	0.753	15.7 ± 3.66	16.2 ± 5.11	0.895	**0.022**
PUFA (%)	7.2 ± 2.46	8.7 ± 4.24	0.274	7.1 ± 3.18	7.8 ± 1.53	**0.033**	0.751
Carbohydrate (%)	50.0 ± 7.00	47.0 ± 11.00	0.326	43.0 ± 10.00	43.0 ± 7.00	0.944	0.130
Dietary fiber (g/day)	13.1 ± 4.76	11.6 ± 4.42	0.242	10.7 ± 5.74	9.3 ± 4.48	0.392	**0.022**
Omega‐6 (g)	11.3 ± 5.07	10.1 ± 6.24	0.263	7.8 ± 6.74	8.3 ± 3.24	0.135	0.445
Omega‐3 (g)	0.8 ± 0.28	1.3 ± 1.21	0.104	1.0 ± 0.36	1.0 ± 0.37	0.750	0.707
Omega‐6/omega‐3	15.3 ± 7.22	10.3 ± 8.54	**0.031**	7.0 ± 3.19	8.9 ± 3.3	0.056	0.581
DTAC	3.9 ± 11.36	1.4 ± 0.86	0.580	5.8 ± 15.74	1.2 ± 0.52	0.138	0.981

*Note*: Independent parameters, Mann–Whitney *U* test; Dependent parameters, Wilcoxon test

Abbreviations: DTAC: dietary total antioxidant capacity, MUFA: monounsaturated fatty acid, PUFA: polyunsaturated fatty acid, SFA: saturated fatty acid

^a^ intra‐group comparison (experimental group, Week 0 and Week 8).

^b^ intra‐group comparison (control group, Week 0 and Week 8).

^c^ inter‐group comparison (experimental and control groups, Week 8).

*p* < 0.05.

**TABLE 6:** Autistic children's micro nutrient intakes before and after nutrition education.

	Experimental group (*n* = 21)			Control group (*n* = 23)			
	Week 0	Week 8	^a^ *p*	Week 0	Week 8	^b^ *p*	^c^ *p*
	Mean±SD	Mean±SD		Mean±SD	Mean±SD		
Vitamin A (µg/day)	592.2 ± 407.19	749.0 ± 712.97	0.428	585.6 ± 451.05	524.3 ± 191.52	0.323	0.991
Vitamin E (mg/day)	10.0 ± 6.28	11.5 ± 7.85	0.414	7.8 ± 6.15	9.2 ± 6.26	0.350	0.318
Vitamin K (µg/day)	69.2 ± 129.58	66.0 ± 126.31	0.753	39.6 ± 18.38	45.7 ± 15.35	0.227	0.072
Thiamin (mg/day)	0.6 ± 0.21	0.5 ± 0.17	0.669	0.6 ± 0.38	0.6 ± 0.33	0.054	0.518
Riboflavin (mg/day)	0.8 ± 0.37	1.0 ± 0.28	0.131	0.8 ± 0.31	0.9 ± 0.19	0.709	0.285
Niacin (mg/day)	15.2 ± 6.88	16.9 ± 7.77	0.385	16.5 ± 7.80	14.7 ± 4.70	0.253	0.353
Vitamin B_6_ (mg/day)	0.8 ± 0.38	0.9 ± 0.33	0.729	0.9 ± 0.28	0.8 ± 0.39	0.552	0.409
Folic acid (µg/day)	151.2 ± 67.97	164.7 ± 76.15	0.678	168.5 ± 90.71	158.1 ± 69.00	0.860	0.860
Vitamin B_12_ (µg/day)	2.7 ± 1.60	3.2 ± 1.68	0.222	2.9 ± 1.54	3.0 ± 0.70	0.843	0.751
Vitamin C (mg/day)	70.6 ± 37.69	74.5 ± 40.18	0.850	64.5 ± 30.91	58.9 ± 31.69	0.743	0.318
Calcium (mg/day)	392.1 ± 160.20	461.0 ± 132.64	0.137	419.6 ± 17.42	496.1 ± 171.84	0.139	0.456
Magnesium (mg/day)	168.4 ± 64.75	160.0 ± 49.6	0.811	172.3 ± 79.85	161.6 ± 69.92	0.629	0.565
Iron (mg/day)	6.3 ± 2.42	5.8 ± 2.07	0.327	6.3 ± 2.28	5.2 ± 1.54	0.087	0.518
Zinc (mg/day)	6.2 ± 2.29	5.8 ± 1.88	0.559	6.3 ± 2.58	6.3 ± 1.92	0.986	0.399

*Note*: Independent parameters, Mann–Whitney *U* test; Dependent parameters, Wilcoxon test

^a^ intra‐group comparison (experimental group Week 0 and Week 8).

^b^ intra‐group comparison (control group, Week 0 and Week 8).

^c^ intergroup comparison (experimental and control group, Week 8).

*p* < 0.05.

## 4 Discussion/Conclusion

Patients with ASD have been shown to be under oxidative stress and to have a greater disposition to neurotoxicity and various disease states including neurodegenerative and neuropsychiatric conditions due to oxidative stress (Griffiths and Levy 2017; Liu et al. 2022). Total serum antioxidant levels decrease in individuals with ASD, causing an increase in the production of oxidative species in the pathology of the disease. Numerous studies have reported GSH as a biomarker of oxidative stress in patients with ASD, with some observing higher levels compared to healthy controls, and others lower (Faber et al. 2019). However, a recent meta‐analysis determined lower GSH and total GSH levels in individuals with ASD than in healthy controls (Chen et al. 2021). In general, GSH levels will decline as oxidative stress increases since the production of reactive oxygen species (ROS) will exceed that of antioxidant molecules. However, GSH production may also rise to reduce increasing oxidative stress severity in the organism (Ghanizadeh et al. 2012). In the current project, GSH levels increased significantly at the end of eight weeks compared to baseline, both in the experimental group in which the families were given nutrition education and in the control group with no such education. Receipt of nutrition education had no significant effect on the control or experimental groups in terms of GSH levels. A previous study detected elevated MDA and 4‐hydroxynonenal levels in the frontal brains of patients with ASD (Frackowiak et al. 2013). This suggests that lipid peroxidation occurs in the brains of patients with ASD. An increase in blood MDA levels has been observed as a typical indication of lipid peroxidation in children with ASD. However, some studies have not detected MDA elevation in the blood of patients with the condition (Al‐Gadani et al. 2009; Essa et al. 2012; Meguid et al. 2011). This inconsistency may derive from the measurement methods employed. MDA is mostly measured using the thiobarbituric acid method (Agarwal and Chase 2002). However, the ELISA method was employed in the present study. No significant difference in MDA levels was determined between the groups (control and experimental, pre‐ and post‐test). While some researchers have reported increased serum SOD levels in children with ASD (Essa et al. 2013), others have reported a decrease (Bambini‐Junior et al. 2011; Afrazeh et al. 2015; Wang et al. 2016), and others have observed no significant difference in SOD activity between ASD patients and healthy controls (Söğüt et al. 2003; Yui et al. 2017). This has raised the question of the use of SOD concentrations as a biomarker in the diagnosis and determining the severity of ASD (Afrazeh et al. 2015). No significant difference in SOD levels was observed between the ASD patients with and without nutrition education in the present study. Researchers have suggested that increased CAT levels in children with ASD may represent a mechanism that develops for eliminating oxidative stress and the damage caused by it (Altun et al. 2018; Yenkoyan et al. 2018). CAT levels rose in both groups in the present study, and there was no significant difference between the two. Research has reported higher 8‐OHdG levels in children with ASD relative to healthy controls (Bilgen Ulgar et al. 2022; Kilicaslan et al. 2019). Another study reported higher urinary 8‐OHdG concentrations among individuals with ASD than in a control group, although the difference was not statistically significant (Ming et al. 2005). 8‐OHdG is the most widely employed parameter for determining DNA damage caused by ROS and has also been linked to mitochondrial dysfunction and metabolic disorders (Kirdeci et al. 2021). In the present study, 8‐OHdG levels increased significantly at the end of the eighth week compared to baseline in the experimental group receiving nutrition education and in the control group. Receipt of nutrition education had no significant effect on either group in terms of 8‐OHdG levels.

Studies have shown an association between ASD and the age of the mother and father. The prevalence of autism is higher among children with older mothers and fathers. A maternal age over 35 and a paternal age over 40 particularly raise the risk of autism (Durkin et al. 2008). The mean age of the mothers of children with autism in this study was 35.5 years, and that of the fathers was 39.41. In addition, there was no significant difference between the experimental and control groups in terms of maternal or paternal ages (*p* > 0.005). The absence of any significant difference between the two groups’ familial characteristics enhances the reliability of this study. The selection of groups with similar characteristics is an important component in determining the effectiveness of the intervention applied (nutrition education). The selection of groups with similar features in this study represents a strength in terms of its evaluation of the efficacy of nutrition education.

The absence of any significant differences between the experimental and control groups in terms of autistic children's demographic and disease‐related characteristics strengthens the reliability of the study. The selection of groups with similar features in this study represents a strength in terms of the ability to evaluate the efficacy of nutrition education without a confounding factor.

Children with ASD have been reported to possess more nutrition problems than their peers (Gray et al. 2023). A previous study on that subject observed an increase in vegetable consumption and the variety of foods consumed in autistic children whose parents received nutrition education (Gray et al. 2023). Another study evaluated the feasibility and efficacy of a 16‐week parental education program for children with ASD and moderate food selectivity and found that such education exhibited positive effects on the nutritional behavior of such children (Sharp et al. 2019). In another study, Sharp et al. reported positive changes in the nutritional behavior of autistic children with a parental training curriculum developed to manage reluctance to eat and low intake in such patients (Sharp et al. 2014). Total BAMBI score elevation indicates greater autism‐specific negative behaviors. Total and subscale scores (limited food variety, food refusal, and features of autism) decreased significantly at the end of the present study (the eighth week) but increased in the control group. Total scores on the children's eating behavior questionnaire, developed to determine children's appetite, also decreased significantly in the experimental group while increasing in the control group (*p* < 0.05), (Table 4). In terms of the questionnaire subscales, food responsiveness, desire to drink, and satiety responsiveness scores decreased in the experimental group, while food responsiveness, emotional overeating, enjoyment of food, satiety responsiveness, slowness in eating, emotional undereating, and fussiness scores increased in the control group (*p* < 0.05) (Table 4). It may therefore be concluded that nutritional education given to patients produced improvements in the eating behaviors of autistic children. The nutrition education given to families is of great importance in children with ASD (Tike Bafra and Kargin 2009). Proper nutrition education and regulation of eating habits commencing from childhood allow children with ASD to live healthier lives. Parents must possess the requisite knowledge for their children to eat adequately and in a balanced manner (Dufault et al. 2024) Existing nutrition problems in children with ASD are a factor with adverse impact on the lives of the family and the child in the long term (Ponchillia et al. 2005; İlik and Sayın 2018). Findings obtained from the three‐day food consumption records of autistic children in a previous study revealed high protein and fat consumption, but inadequate vitamin C and calcium consumption (Meguid et al. 2015). Plaza‐Diaz et al. reported higher energy and fat consumption in children with ASD compared to healthy. Following nutrition education in the present study, daily energy, SFA, carbohydrate, omega‐6, and dietary TAC intake decreased among the children with ASD in the experimental group, while their protein, fat, MUFA, PUFA, and omega‐3 intake rose. However, these findings were not statistically significant (*p* > 0.05). Omega‐6/omega‐3 values decreased significantly by the eighth week following nutrition education (*p* < 0.05). The control group's PUFA intake also increased (*p* < 0.05). MUFA intake was lower among the children in the experimental group than in the control group, while dietary fiber intake was higher (*p* < 0.05). No statistically significant difference in micronutrient intake was observed before and after nutrition education in the children from either group (*p* > 0.05). These findings all show that nutrition education given to families helps children with ASD acquire healthy nutrition and eating habits, prevents nutrient deficiencies, alleviates nutrition‐related symptoms, ensures healthy growth and development, increases the quality of life of families, and reduces stress factors.

The principal limitations of this study are multifaceted and must be considered when interpreting the findings. First, the duration of the nutrition education intervention, which lasted only eight weeks, may have been insufficient to observe significant changes in antioxidant and oxidant parameters. These parameters often require longer periods of consistent dietary and lifestyle modifications to reflect measurable improvements, and the short intervention duration likely limited the study's ability to capture such changes.

Second, there were challenges related to the full implementation of the nutrition education program by the children. Despite the structured guidance provided during the sessions, adherence to the recommendations heavily depended on the families' ability to support and reinforce these changes at home. Factors such as family routines, socioeconomic status, and parental involvement may have affected the extent to which children could apply the knowledge and practices shared during the program. These barriers to adherence could have diluted the potential impact of the intervention.

Lastly, the relatively low number of participants in the study presents another significant limitation. A small sample size reduces the statistical power of the study, making it more challenging to detect meaningful differences or draw generalizable conclusions. In addition, with a limited number of patients, there is an increased risk of variability or bias influencing the results, further impacting the reliability of the findings.

Future studies could address these limitations by extending the duration of nutrition education programs, incorporating strategies to enhance family engagement and support, and increasing the sample size to strengthen statistical robustness and the generalizability of outcomes.

## Author Contributions

**Diler Us altay**: Funding acquisition, methodology, supervision, writing – review and editing, investigation, conceptualization, writing – original draft, project administration. **Erman Esnafoglu**: Conceptualization, methodology, supervision, data curation. **Emine Kocyigit**: Conceptualization, supervision, validation, writing – review and editing, investigation. **Duygu Mataraci değirmenci**: Conceptualization, writing – review and editing, validation, supervision, investigation. **Tevfik Noyan**: Conceptualization, investigation.

## Ethics Statement

This study was reviewed and approved by the Institutional Review Board at the University of Ordu, Turkiye, and was performed under the principles of the Declaration of Helsinki. Ethics committee approval was received from the Ordu University Local Ethical Committee Under Reference No: 2023/206

## Conflicts of Interest

The authors declare no conflicts of interest.

### Peer Review

The peer review history for this article is available at https://publons.com/publon/10.1002/brb3.70504

## Data Availability

The data that support the findings of this study are available from the corresponding author upon reasonable request.
